# PSG Gene Expression Is Up-Regulated by Lysine Acetylation Involving Histone and Nonhistone Proteins

**DOI:** 10.1371/journal.pone.0055992

**Published:** 2013-02-13

**Authors:** Soledad A. Camolotto, Ana C. Racca, Magali E. Ridano, Susana Genti-Raimondi, Graciela M. Panzetta-Dutari

**Affiliations:** Centro de Investigaciones en Bioquímica Clínica e Inmunología (CIBICI-CONICET), Departamento de Bioquímica Clínica, Facultad de Ciencias Químicas, Universidad Nacional de Córdoba, Córdoba, Argentina; Fudan University, China

## Abstract

**Background:**

Lysine acetylation is an important post-translational modification that plays a central role in eukaryotic transcriptional activation by modifying chromatin and transcription-related factors. Human pregnancy-specific glycoproteins (PSG) are the major secreted placental proteins expressed by the syncytiotrophoblast at the end of pregnancy and represent early markers of cytotrophoblast differentiation. Low PSG levels are associated with complicated pregnancies, thus highlighting the importance of studying the mechanisms that control their expression. Despite several transcription factors having been implicated as key regulators of *PSG* gene family expression; the role of protein acetylation has not been explored.

**Methodology/Principal Findings:**

Here, we explored the role of acetylation on *PSG* gene expression in the human placental-derived JEG-3 cell line. Pharmacological inhibition of histone deacetylases (HDACs) up-regulated PSG protein and mRNA expression levels, and augmented the amount of acetylated histone H3 associated with *PSG* 5′regulatory regions. Moreover, *PSG5* promoter activation mediated by Sp1 and KLF6, via the core promoter element motif (CPE, −147/−140), was markedly enhanced in the presence of the HDAC inhibitor trichostatin A (TSA). This effect correlated with an increase in Sp1 acetylation and KLF6 nuclear localization as revealed by immunoprecipitation and subcellular fractionation assays. The co-activators PCAF, p300, and CBP enhanced Sp1-dependent *PSG5* promoter activation through their histone acetylase (HAT) function. Instead, p300 and CBP acetyltransferase domain was dispensable for sustaining co-activation of *PSG5* promoter by KLF6.

**Conclusions/Significance:**

Results are consistent with a regulatory role of lysine acetylation on *PSG* expression through a relaxed chromatin state and an increase in the transcriptional activity of Sp1 and KLF6 following an augmented Sp1 acetylation and KLF6 nuclear localization.

## Introduction

Histone post-translational modifications have been shown to be crucial for programmed gene expression during several events of development in eukaryotes, including placental development and functioning [1]. Histone covalent modifications include lysine acetylation, lysine and arginine methylation, serine and threonine phosphorylation, lysine ubiquitination, lysine sumoylation, and glutamic acid poly-ADP-ribosylation [2]. Among them, histone tail acetylation has been strongly correlated with transcriptional activation [3]. This reversible modification is carried out by two classes of enzymes, histone acetyltransferases (HATs) and histone deacetylases (HDACs) [4]. cAMP-response-element-binding protein (CREB)-binding protein (CBP), p300, and p300/CREB-binding protein-associated factor (PCAF) are among the several HATs described so far. They act as transcriptional co-activators with the capability to interact with a wide range of transcription factors and integrate signals from different pathways, modify histones, and remodel chromatin. Remarkably, HATs and HDACs are not exclusively targeted towards histones. Some of the nonhistone targets are transcription factors, chaperones, basal transcriptional machinery components, signal transducers, hormone receptors, and cytoskeleton proteins [5], [6]. Dynamic acetylation of histone and nonhistone proteins can be selectively modulated by HDAC inhibitors (HDACis), thereby regulating gene transcription by affecting chromatin assembly and/or modifying protein–protein interactions, protein stability, DNA-binding capability, transcriptional activity, and/or nuclear localization of specific protein factors [7].

The ubiquitously expressed transcription factors Sp1 and Krüppel-like factor 6 (KLF6) are well known molecular targets of CBP, p300, and PCAF HAT function as well as of HDACs, in several biological systems [8]–[12]. Sp1 and KLF6 have been described as important regulators for normal placental development and formation [13], [14] and they have also been associated with the transcriptional control of placental specific genes such as *17 β-hydroxysteroid dehydrogenase* [15], *human chorionic gonadotropin β subunit* (*hCGβ)* [16], and *pregnancy-specific glycoprotein 3* and *5 (PSG3* and *PSG5)* [17]–[19].

PSGs constitute the major group of secreted proteins synthesized by the placental syncytiotrophoblast, reaching 200–400 mg/L in maternal serum at the end of normal gestation [20]. Although their functions have not been fully established, several lines of evidence suggest they are essential for the maintenance of a normal pregnancy. Indeed, low PSG levels are associated with a poor pregnancy outcome [21], [22]. In addition, they promote alternative macrophage activation, which correlates with a shift from inflammatory Th1- to anti-inflammatory Th2-mediated immunological responses *in vitro* and *in vivo* [23]–[25]. Current data also suggest that PSGs play an important role in the process of vasculature establishment at the maternal-fetal interface ensuring feto-placental blood supply [26], [27]. The measurement of PSG serum levels was proposed as a useful biomarker to monitor and diagnose gestational pathologies more than thirty years ago [28], [29]. Human PSGs are encoded by 11 genes clustered within 700-kilobases on chromosome 19q13.2, which share above 90% nucleotide sequence identity [30]. *PSG* gene promoters are highly homologous lacking common minimal consensus promoter sequences such as TATA-box, initiator elements or long pyrimidine-rich GC regions [31], [32]. We have previously demonstrated that *PSG5* promoter activity is largely dependent on a core promoter element (CPE, CCCCACCC) conserved in all *PSG* genes [33]. This sequence mediates *PSG5* transcriptional activation by Sp1 and KLF6 [18], [19]. In addition, KLF4 and Retinoid X receptor alpha, as well as the RARE and GABP consensus-binding sites located at the proximal promoter are involved in *PSG* gene transcription [17], [34], [35]. However, the remarkable increase in PSG biosynthesis associated with villous trophoblast differentiation strongly suggests the contribution of other regulatory mechanisms.

The aim of this work was to explore whether lysine acetylation of both histone and specific transcription factors is involved in *PSG* gene expression control. Here, we demonstrate that *PSG* gene expression is markedly up-regulated when HDACs are inhibited. Gene activation correlates with a higher level of acetylated histone H3 associated with the *PSG* 5′ flanking regions. In addition, *PSG5* induction by Sp1 and KLF6 transcription factors is potentiated by TSA treatment, which also increases Sp1 acetylation and KLF6 nuclear localization. Finally, Sp1-mediated activation of the *PSG5* promoter is further induced by CBP, p300, and PCAF in a HAT-dependent manner, whereas CBP and p300 exert their function as KLF6 co-activators through a mechanism independent of their HAT activity.

## Materials and Methods

### Culture Conditions

Human placental-derived JEG-3 cell line (ATCC, HTB-36), obtained from the American Type Culture Collection (ATCC, Rockville, USA), was maintained in DMEM supplemented with 10% fetal bovine serum, 100 U/ml penicillin, and 100 µg/ml streptomycin (Invitrogen), at 37°C in 5% CO_2_. For treatments, cells were plated at a density of 1×10^6^ cells/10 cm plates. After overnight incubation, cells were treated for 18 h with several concentrations of NaBu, TSA (Sigma) or with their respective solvent vehicles, H_2_O or 0.015% DMSO.

### MTT Assay

After establishing the adequate cell density for viability/cytotoxicity assay, 1×10^4^ JEG-3 cells/well were plated in a 96-well plate. Twenty four hours later, cells were incubated with NaBu or TSA. After treatment, 10 µl (5 mg/ml) of 3-(4, 5-dimethlthiazol-2-yl)-2, 5-diphenyl-tetrazolium bromide (MTT) was added to each well and cells were incubated for 2.5 h at 37°C. Then, the medium was carefully removed, formazan crystals were dissolved for 5 min in 100 µl of DMSO/well, and absorbance was measured at 570 nm. Cell viability was established as 100% in the control condition where the cells were incubated with H_2_O or 0.015% DMSO.

### Reverse Transcription and Quantitative Real-time PCR

Total RNA from 6-well plates was extracted with TRIzol (Invitrogen) reagent, as recommended by the manufacturer. One microgram of extracted RNA, 25 ng of random hexamers (Invitrogen), 20 U of RNAsin (Promega), and 200 U of murine leukemia virus reverse transcriptase (Promega, Madison, WI, USA) were used to synthesize cDNA.

Total PSG transcript levels were quantified by real-time RT-PCR in an ABI 7500 Sequence Detection System (Applied Biosystems) using the reaction conditions and primer pair previously described [34]. The primers recognize all the *PSG* gene family member transcripts and the amplicons obtained are of identical length. Data are presented as fold change in *PSG* gene expression normalized to peptidylprolyl cis-trans isomerase A (PPIA) expression and relative to non-treated cell cultures selected as the calibrator condition. Each sample was analyzed in triplicate.

### Chromatin Immunoprecipitation (ChIP) Assay

ChIP analyses were carried out according to the ChIP assay kit (Upstate Biotechnology) manufacturer’s instructions with minor modifications. Briefly, cells were cross-linked with 1% formaldehyde at room temperature for 10 min and subsequently, the cross-linking was blocked with 125 mM glycine for 5 min. Cells were lysed in lysis buffer supplemented with protease inhibitor cocktail (Sigma), followed by sonication (Sonics Vibra Cell, USA), and pre-clearing with salmon sperm DNA/protein A agarose. The histone/DNA complexes were incubated overnight at 4°C with 2 µg of polyclonal anti-acetyl-H3 (Upstate Biotechnology) antibody. As immunoprecipitation specificity controls, cell lysates were incubated with 2 µg of polyclonal anti-PSG (A0131, Dako) or without antibody. The immunocomplexes were centrifuged at 12000 rpm and supernatants were precipitated with salmon sperm DNA/protein A agarose at 4°C for 4 h. Complexes were serially washed and then, DNA was purified using 200 µl of 10% p/v Chelex 100 resin (BIORAD). The samples were boiled for 10 min, treated with proteinase K (10 mg/ml) at 55°C for 1 h, and boiled for additional 10 min to inactivate the protease activity. Samples were centrifuged at high speed and the supernatants were recovered.

The amplification reactions were carried out under not saturating conditions using 0.25 mM of each For and Rev primer, annealing temperature of 55°C and 5 mM Mg^2+^ concentration. The primers employed and amplicon sizes are indicated in Table 1.

**Table 1:** Primer sequences used in conventional PCR for ChIP assays and their corresponding amplification product sizes.

Gene	Primer name	Sequence (5′- 3′)	Product size (bp)
***PSG locus***			
	PSG -178 For	attgctagcgagaggaggggacagagaggt	129 bp
	PSG -49 Rev	agagctcgagagaaacttcctgagcacggc	
***PSG locus***			
	PSG -970 For	ccaggctccccctcctgcgtctcaa	487 bp
	PSG -551 Rev	ttaaccccattgtgctgtgggtgagctgtgtg	
***PSG locus***			
	PSG -1463 For	ctgctccatctagactgttctctggg	448 bp
	PSG -977 Rev	caggggttcagagcctggagagatt	
***GAPDH***			
	GAPDH For	tactagcggttttacgggcg	166 bp
	GAPDH Rev	tcgaacaggaggagcagagagcga	
***PSG5***			
	PSG5 coding region For	caagtcacgattgaagccct	300 bp
	PSG5 coding region Rev	tactcctctagtcctatcacctcg	

A transcriptionally active euchromatic region corresponding to a *GAPDH* promoter sequence and a *PSG5* coding region (+234/+533) were used as acetyl H3-DNA immunoprecipitation positive and negative controls, respectively.

### Reporter Constructs

The recombinant promoter-luciferase constructs containing different sizes of *PSG3* and *PSG5* promoters and their 5′ regulatory regions were obtained as previously described [34]. The XB3-luc reporter construct was obtained by amplification of the *PSG3* regulatory region (positions -1463/−49) and cloning into the *Nhe*I and *Bgl*II sites of the pGL3 basic vector (Promega). The CPEmutUB5luc reporter plasmid was obtained from the UB5-luc construct by PCR-directed mutagenesis of the CPE element (5′ CCCCACCCAT 3′ to 5′ CCCCgatatc 3′).

### Transient Transfection and Luciferase Activity Assay

For promoter activity assays, JEG-3 cells seeded at a density of 1×10^5^ per well in 24-well plates were cultured for 24 h and transfected using the optimized conditions described in reference [34]. Medium was completely replaced 4 h post-transfection and cells were treated with 150 nM TSA for 18 h before harvesting. For co-transfection experiments JEG-3 cells were transfected with 2 µl Lipofectamine 2000 (Invitrogen) and 850 ng of total DNA using 500 ng of the indicated *PSG* reporter plasmid and 150 ng of each expression vector in the combinations indicated in figure legends. The expression vectors pCI-PCAF Flag and pCI-PCAFΔHAT Flag [36], pCMV-CBP HA and pCMV-CBPΔHAT HA were gently supplied by Dr. Greer, pCMVβ-p300 HA and pCMVβ-p300 DI1485AL HA were a gift of Dr. Hecht [37], and pXJ-KLF6 [38] and pCMV-Sp1 were kindly provided by Dr. Bocco. The corresponding empty vectors (pCMV, pCI and pXJ-41) were used in control transfections and to adjust the total amount of DNA when necessary. After 48 h post-transfection, cells were collected, lysed in lysis buffer (Promega), and luciferase activity was measured using the Luciferase Assay System (Promega) on a GloMax-Multi Detection System (Promega). Luciferase activity in each sample was normalized to protein levels determined by the Bradford method.

### Immunofluorescence Staining

JEG-3 cells were cultured on cover slips using complete medium with the addition of TSA or NaBu as described above. Immunofluorescence assays were performed as previously reported [19]. The following polyclonal rabbit primary antibodies were used: anti-PSG (A0131, Dako) 1/100, anti-KLF6 (R-173, Santa Cruz Biotechnology) 1/50, anti-Sp1 (H225, Santa Cruz Biotechnology) 1/200, and anti-acetyl-H3 (Upstate) 1/500. Cells were incubated with green Alexa Fluor 488-conjugated donkey anti-rabbit IgG (Molecular Probes, Inc., Eugene, OR) at 1/720 final dilution. Nuclei were counterstained with Höechst 33258 for 15 min. The antibody incubations were performed in a humidity chamber for 1 h at 37°C. Cells were examined under an epifluorescence microscope (Nikon Eclipse TE2000-U, USA) and images were collected with appropriate filters at the magnification indicated in figure legends.

### Western Blot Analysis

Cells were harvested in Laemmli sample buffer after 48 h of HAT co-activator overexpression or after 18 h of TSA treatment and then subjected to protein expression analysis. Western blot assays were performed as described earlier [34]. The following primary antibodies were employed: polyclonal rabbit anti-Flag (Sigma-Aldrich) 1/2500, monoclonal mouse anti-HA (Abcam) 1/1000, polyclonal anti-acetyl-H3 (Upstate) 1/6000, polyclonal anti-Sp1 1/2000, monoclonal mouse anti-KLF6 (clone 2c11, whose specificity was previously determined [38]) 1/10000, polyclonal rabbit anti-PSG (A0131, Dako) 1/500, monoclonal mouse anti-GAPDH (4300 Ambion) 1/1000, polyclonal goat anti-Ku80 (sc-1484 Santa Cruz) 1/1000, and monoclonal mouse anti-β-actin (Sigma-Aldrich) 1/2000.

### Culture Supernatant Protein Detection

To determine PSG protein secretion in culture supernatants, JEG-3 cells were grown and treated as described before. The supernatants of DMSO- and TSA-treated cells were recovered after centrifugation at 10000 rpm for 10 min at 4°C, and subjected to western blot analysis to detect secreted PSG protein. Ponceau staining was used as loading normalizer.

### Immunoprecipitation Assay

JEG-3 cells were transiently transfected with 620 ng pCMV-Sp1 or pXJ-KLF6. Thirty hours later, cells were treated with or without 150 nM TSA for 18 h. Cells were washed with cold 1X PBS, harvested in 500 µl non-denaturizing lysis buffer (20 mM Tris-HCl pH 8, 1% NP40, 10% glycerol, 137 mM NaCl, 2 mM EDTA, 1 mM PMSF, and protease inhibitor cocktail), and then incubated at room temperature for 30 min with rotation. Protein concentration in the supernatant was measured using the Bradford assay. Two hundred micrograms of total protein extracts from each cell condition were incubated overnight at 4°C in an orbital rotor with 6 µg of rabbit anti-Sp1 antibody or 6 µg of anti-KLF6 antibody mix (2 µg polyclonal R-173 and 4 µg monoclonal clone 2c11) previously cross-linked to 2 µl of protein G Mag Sepharose beads (GE Healthcare Life Sciences). The immunocomplexes were washed once with TBS buffer (50 mMTris-HCl pH7.5, 150 mM NaCl), eluted in 0.1 M glycine-HCl buffer (pH 2.5), and then neutralized with 1 M phosphate buffer (NaPO_4_, pH 8). Laemmli sample buffer (5X) was added to each eluted fraction and boiled for 5 min. Proteins were then separated by SDS-PAGE, transferred onto nitrocellulose Hybond-ECL (Amersham Bioscience) membranes, blocked with 5% non-fat milk in PBS-Tween 20 (0.1%), and probed with polyclonal anti-acetyl lysine antibody (Millipore, AB3879) at 1/1500 dilution. The membranes were stripped and re-probed with anti-Sp1 (1/2000) or anti-KLF6 (monoclonal clone 2c11, 1/10000) antibodies. Bands were revealed by enhanced chemiluminescence detection system (SuperSignal West Pico; Pierce) and visualized by exposing to Kodak T-Mat G/RA films.

### Subcellular Fractionation

JEG-3 cells treated with 150 nM TSA or DMSO were washed three times with 1X PBS and incubated for 15 min on ice with 500 µl lysis buffer (50 mM Tris-HCl pH 7.5, 137.5 mM NaCl, 10% glycerol, 1 mM sodium vanadate, 50 mM NaF, 10 mM Na_4_P_2_O_7_, 5 mM EDTA, and protease and phosphatase inhibitor cocktails) containing 0.5% Triton X-100. The lysates were centrifuged at 13000 rpm for 15 min at 4°C to obtain the membrane/cytoplasmic fraction in the supernatant. The nuclear pellets were rinsed once with lysis buffer, then resuspended in 150 µl 0.5% SDS-lysis buffer, and sonicated for 5 seconds. Lysates were pre-cleared by centrifugation at 13000 rpm for 15 min at 4°C. Laemmli sample buffer (5X) was added to nuclear and membrane/cytoplasmic fractions, boiled for 10 min, and then subjected to western blot assay. The presence of Ku80 and GAPDH exclusively in the nuclear and membrane/cytoplasmic fractions, respectively, confirmed the cell fractionation success. In addition, they were used as loading control of each subcellular fraction.

### Statistical Analysis

Pair-wise comparison between groups was evaluated with a 2-tailed Student’s t test or one-way ANOVA between multiple groups followed by Fisher’s test to determine a statistical difference (Infostat Software, http://www.infostat.com.ar). A value of p≤0.05 was considered statistically significant.

## Results

### PSG Protein and mRNA Expression is Stimulated by HDAC Inhibitors

**Figure 1:**
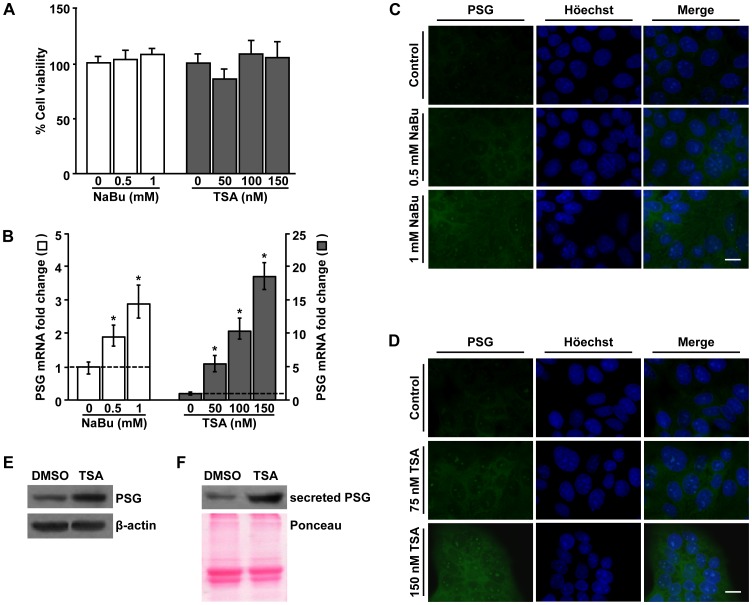
Total PSG protein and mRNA expression is stimulated by HDAC inhibitors. A) JEG-3 cells were cultured in the presence of the indicated concentrations of NaBu (white bars), TSA (grey bars) or vehicle alone (0, control condition) for 18 h. MTT assay was used to determine cell survival. Results represent the mean ± SEM of three independent experiments performed in sixtuplicates and are shown as percentage respect to the non-treated cultures established as 100%. No statistically significant differences (p>0.05) compared to the control condition were detected. B) Total PSG mRNA levels were determined by real time RT-PCR (ABI 7500, Applied Biosystems) in JEG-3 cells cultured in the presence of the specified concentrations of NaBu (white bars) or TSA (grey bars) and in control cultures (0). Results were normalized to PPIA and expressed according to the 2^–ΔΔCt^ method using as calibrator the mRNA level obtained from the corresponding control cultures. Data are presented as mean ± SEM of four independent experiments performed in triplicates. Significant differences were set at * p≤0.05, respect to control. C, D) Immunofluorescence of PSG (green, left panels) and nuclear staining with Höechst (blue, middle panels) in JEG-3 cells after treatment with the indicated amounts of NaBu (C), TSA (D) or vehicle (control). Right panels: merged images. Bar = 10 µm. Original magnification: 1000X. E, F) Western blot detection of PSG protein in JEG-3 cell total extracts (E) and secreted PSG levels in culture supernatants (F) after 150 nM TSA or DMSO exposure. β-actin (for PSG cellular content) and Ponceau staining (for secreted PSG) were used as loading normalizers. A representative experiment of two independent assays is shown.

To investigate whether acetylation is involved in the activation of *PSG* gene expression we used the human JEG-3 cell line. These cells retain features of placental trophoblasts, synthesize several placental hormones and enzymes, and represent an established model for trophoblast studies [34], [39], [40]. JEG-3 cells were treated with the HDACis TSA or NaBu, at a range of concentrations that did not alter cell viability (Fig. 1A). Under these experimental conditions, PSG transcript levels increased when compared to non-treated control cultures, as determined by real time RT-PCR (Fig. 1B). The induction of PSG mRNA was much higher with TSA than NaBu, which is in line with the reported stronger inhibition of HDACs by TSA [41]. In addition, enhanced PSG protein staining was detected by immunofluorescence after 18 h of incubation with either NaBu (Fig. 1C) or TSA (Fig. 1D). The highest induction of PSG protein and mRNA levels was observed at 150 nM TSA, condition that was selected for conducting the following experiments. In this condition, PSG protein levels (Fig. 1E) increased and PSG secretion (Fig. 1F) was also up-regulated as revealed by western blot detection in total cell lysates and culture supernatants, respectively.

### TSA Treatment Increases Acetylation of Histone H3 Associated with *PSG* Gene Promoters

**Figure 2:**
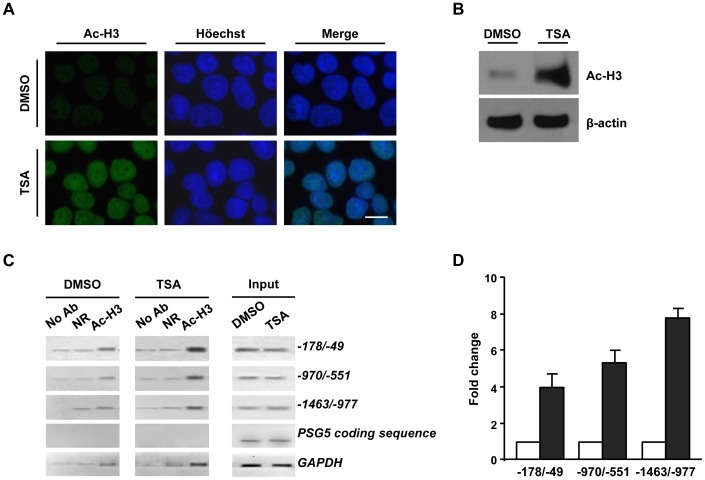
TSA treatment increases acetylation of histone H3 associated with *PSG* gene promoters. JEG-3 cells were exposed to 150 nM TSA or 0 nM TSA (DMSO 0,015%, non-treated controls) for 18 h. A) Epifluorescence immune detection of acetyl H3 (Ac-H3) protein (green, left panels) and Höechst counterstaining (blue, middle panels). Merged images are shown on the right panels. Scale bar: 10 µm. Original magnification: 1500X. B) Western blot assay to detect whole cell content of acetyl H3 (Ac-H3) in protein extracts from JEG-3 cells treated with DMSO or TSA. β-actin protein was used for normalization of protein loading. A representative experiment of three independent assays is shown. C) ChIP analyses. TSA- and DMSO-treated JEG-3 cells were fixed with formaldehyde. DNA-protein complexes were sonicated and immunoprecipitated using anti-acetyl-H3 (Ac-H3), anti-PSG (as non related antibody, NR) or without antibodies (No Ab). Input and recovered DNA samples were amplified by conventional PCR employing primer pairs flanking positions −178/−49, −970/−551, and −1463/−977 of the regulatory region of *PSG* genes (positions are indicated relative to the *PSG5* gene). A *PSG5* coding sequence and a transcriptionally active euchromatin region of the *GAPDH* promoter were used as negative and positive immunoprecipitation controls, respectively. Results are representative of duplicated PCR reactions from two independent ChIP assays. D) Densitometric quantification of amplified products of the *PSG* gene regulatory regions normalized to the corresponding input is shown after subtraction of No Ab and NR amplification. Results are presented as mean ± SD and referred as fold change respect to DMSO control condition (arbitrarily defined as 1).

HDACis have been described to induce a global increase of histone acetylation in the nucleus [42], however, it is well documented that their effects on gene expression are not global but they rather alter the expression of a few specific genes (1–7%) with a comparable number of repressed and derepressed genes [43]–[45]. High throughput analysis have demonstrated that acetyl H2AK7, H3K9, H3K14, H3K18, H4K5, and H4K12 are found principally enriched in the 5′ regulatory region of active genes, where they create an accessible chromatin domain. Particularly, acetylation of histone H3K9/14 has been proposed as a signature of active transcription [46]–[48]. Therefore, we investigated whether the increase in *PSG* expression correlated with an augmented level of acetylated H3K9/14 at the 5′ regulatory region of *PSG* genes. First, we confirmed a global increase of acetylated H3 level in JEG-3 cells treated with 150 nM TSA compared to control cells by immunofluorescence and western blot analysis carried out using the specific anti-acetyl-H3 antibody that recognizes acetyl H3K9 and K14 (Fig. 2A and B). Next, ChIP assays were performed on chromatin isolated from TSA-treated or control JEG-3 cells immunoprecipitated with the anti-acetyl-H3 antibody. The amplification of three conserved DNA fragments in the promoter and 5′ regulatory region of all *PSG* genes clearly demonstrated that they were enriched in acetylated histone H3 in TSA-treated cells (Fig. 2C and D). As expected, no or faint amplification was detected for the *PSG5* coding sequence, as well as for all the samples incubated with the non-related antibody or without antibody. Instead, the *GAPDH* promoter sequence was clearly amplified in the anti-acetyl-H3 immunoprecipitated DNA (Fig. 2C).

In summary, these results indicate that TSA treatment correlates with an increased acetylation of histone H3 associated with the *PSG* gene promoter and 5′ proximal regulatory regions.

### TSA Treatment Markedly Induces *PSG3* and *PSG5* Promoter Activities

**Figure 3:**
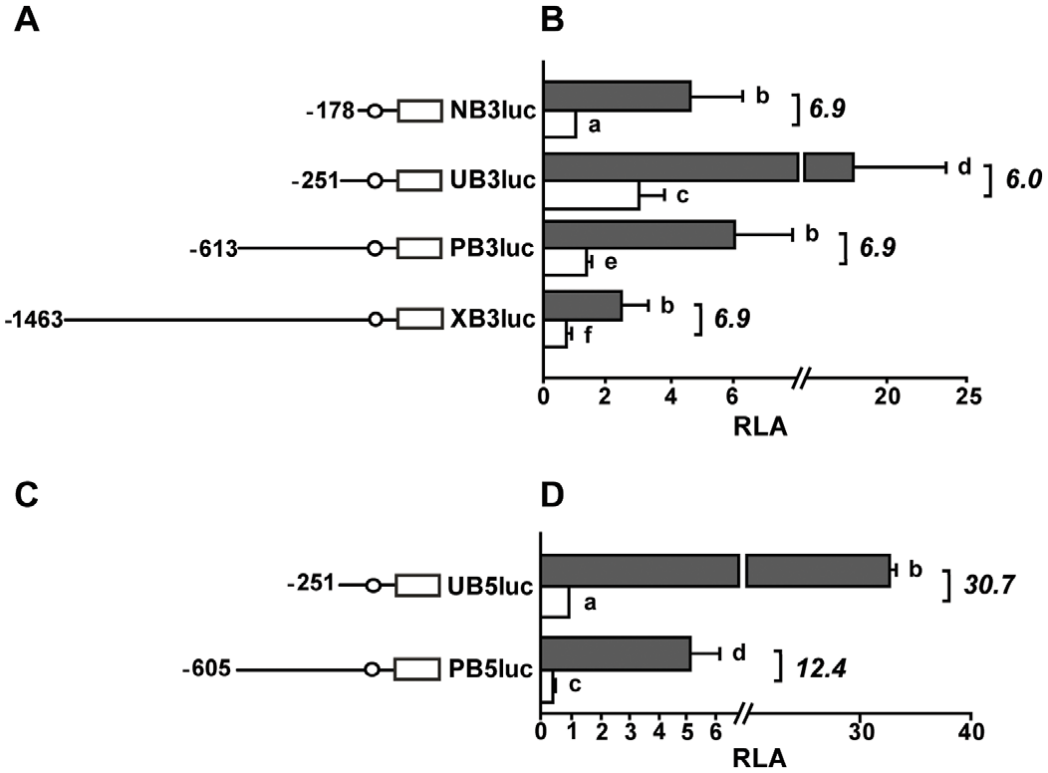
Induction of *PSG3* and *PSG5* promoter activity in TSA-exposed JEG-3 cells. Scheme of reporter constructs bearing the 5′ regulatory region of *PSG3* (A) and *PSG5* (C) genes. Circles depict the CPE regulatory element. Luciferase activity was determined in JEG-3 cells incubated with 150 nM TSA (grey bars) or DMSO (white bars). Results are expressed as relative luciferase activities (RLA) referred to the promoter activity of NB3luc (B) or UB5luc (D) construct in DMSO control cultures, arbitrarily set as 1. Data are shown as mean ± SEM of four independent experiments performed in triplicates. Italic bold numbers represent fold increase in luciferase activity of each construct in TSA-exposed cells relative to the control ones. Data were subjected to analysis of variance (one-way ANOVA) followed by Fisher's test to determine significant statistical differences (p≤0.05). Different letters on the right side of each bar indicate statistically different values.

As mentioned before, HDAC inhibition can modulate gene expression not only through the induction of a local chromatin conformation change, but also modifying the function of nonhistone proteins. Thus, to further investigate the relationship between HDAC inhibition and transcriptional up-regulation of *PSG* genes, we examined whether TSA could activate *PSG3* and *PSG5* promoters. JEG-3 cells were transfected with luciferase reporter plasmids containing different fragments of the 5′ regulatory regions and then, they were treated with TSA. A notable increase in the reporter activity of all *PSG3* (Fig. 3A and B) and *PSG5* (Fig. 3C and D) promoter constructs was detected. *PSG3* reporter constructs reached activities up to 6-7-fold higher in cells cultured with TSA than controls (Fig. 3B), while UB5luc and PB5luc constructs were activated 30.7- and 12.4-fold (Fig. 3D), respectively, in the presence of TSA compared to the control condition. These data strongly suggest that TSA induces *PSG3* and *PSG5* expression at the transcriptional level and reveal that proximal promoter sequences are potentially involved in the response to HDAC inhibition.

### TSA Enhances Sp1- and KLF6-induced *PSG5* Promoter Activation through the CPE Element

**Figure 4:**
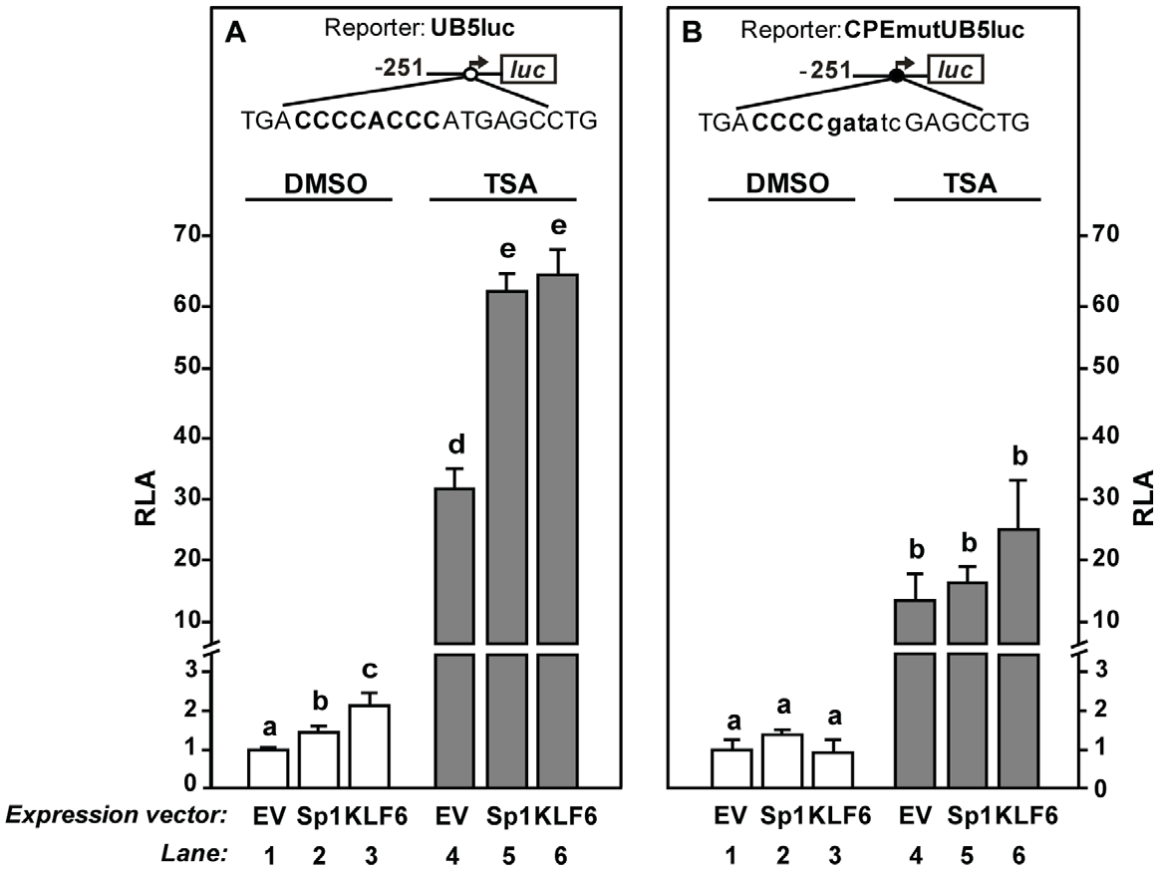
TSA enhances Sp1- and KLF6-induced *PSG5* promoter activity through the CPE element. Cells co-transfected with the UB5luc (A) or CPEmutUB5luc (B) construct and the Sp1 (lanes 2 and 5), KLF6 (lanes 3 and 6) or empty (lanes 1 and 4) expression plasmids were cultured in the presence of DMSO (lanes 1–3) or 150 nM TSA (lanes 4–6). Data represent relative luciferase activity (RLA) referred to that of each reporter construct co-transfected with the corresponding empty vector (EV) and treated with DMSO. Results are shown as mean ± SEM of triplicates of two to four independent assays. Different letters on the bars indicate statistically different values (p≤0.05, Fisher test following one-way ANOVA). CPE binding sequence (uppercase bold letters) and mutated nucleotides (lowercase letters) are depicted.

The CPE motif is an important *cis*-regulatory element present in all *PSG* gene proximal regulatory sequences. This motif is involved in *PSG5* promoter activation and is recognized by Sp1 and KLF6, as previously demonstrated by luciferase reporter, EMSA and supershift assays [18], [19], [49]. Therefore, we analyzed whether TSA could modulate Sp1 and KLF6 effect on *PSG5* promoter activity. In the absence of this HDACi (DMSO, control condition) the UB5luc construct activity was induced near 1.5- and 2.0-fold by Sp1 and KLF6 overexpression, respectively (Fig. 4A, lanes 2 and 3 *vs* 1), confirming previous results [18], [19]. UB5luc reporter activity was increased almost 60-fold in cells transfected with Sp1 or KLF6 and treated with TSA compared to control basal condition (Fig. 4A, lanes 5 and 6 *vs* 1) and near 2-fold respect to the induction provoked by TSA alone (Fig. 4A, lanes 5 and 6 *vs* 4). These observations are consistent with a potentiation of Sp1- and KLF6-dependent transactivation of the UB5luc reporter in the presence of TSA. In contrast, reporter activity of the CPEmutUB5luc construct was not stimulated by Sp1 or KLF6 transcription factors (Fig. 4B, lanes 2 and 3 *vs* 1), and the synergistic activation by TSA was lost (Fig. 4B, lanes 5 and 6 *vs* 4). Although TSA was able to augment the reporter activity of the CPEmutUB5luc construct (Fig. 4B, lane 4 *vs* 1), the wild type promoter construct was induced near 31-fold while the CPE mutated one was stimulated only about 10-fold (p≤0.05). These results indicate that TSA-mediated *PSG5* promoter stimulation partially depends on the CPE motif, suggesting that other regulatory sequences located in the −254/−49 *PSG5* promoter region are also involved.

In summary, these data indicate that TSA potentiates Sp1- and KLF6-induced *PSG5* promoter activation through a functional CPE binding site.

### TSA Stimulates Sp1 Acetylation and KLF6 Nuclear Localization

**Figure 5:**
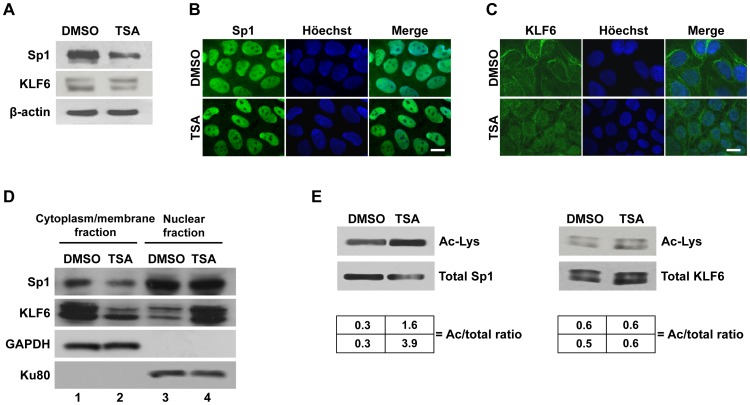
TSA stimulates Sp1 acetylation and KLF6 nuclear localization. A) Protein extracts prepared from JEG-3 cells exposed to TSA or DMSO (control condition) for 18 h were subjected to western blot analysis using anti-Sp1 (upper panel), anti-KLF6 (middle panel), and anti-β-actin (lower panel) antibodies. Representative blots are shown of at least three independent experiments with similar results. B, C) Immunofluorescence analyses of endogenous Sp1 and KLF6 (green signal, left panels) distribution in DMSO-cultured JEG-3 cells (upper panels) and TSA-treated cells (lower panels). Nuclei were counterstained with Höechst dye (blue signal, middle panels). Merged images are shown on the right panels. The images presented are representative of three independent experiments. Scale bar = 10 µm. Original magnification = 1000X. D) Western blot analysis of the endogenous Sp1 and KLF6 protein content detected in the cytoplasmic/membrane (lanes 1 and 2) and nuclear (lanes 3 and 4) fractions of JEG-3 cells treated with DMSO (lanes 1 and 3) or TSA (lanes 2 and 4). GAPDH and Ku80 protein levels were used as loading and fractionation controls. One representative experiment of four independent assays with consistent results is shown. E) Immunoprecipitation and western blot assays were performed to determine the acetylation state of overexpressed Sp1 and KLF6 in JEG-3 cells treated or not with TSA. Whole cell lysates were first precipitated with anti-Sp1 or a mix of anti-KLF6 antibodies and then subjected to western blotting with anti-acetyl lysine antibody (Ac-Lys). The same amount (30 µg) of immunoprecipitated proteins were loaded in each lane. The blots of one representative of two different experiments are shown. The ratio of acetylated (Ac) to total Sp1 or KLF6 of each independent experiment is indicated below each band.

Sp1 and KLF6 transcription factors have been described as molecular targets of lysine acetylation/deacetylation [8]–[12]. Thus, we decided to evaluate whether TSA modulates Sp1 and KLF6 function through modifications in their acetylation, localization, and/or expression in trophoblast cells. To this end, JEG-3 cells were treated with TSA or DMSO (control) for 18 h and the endogenous transcription factor expression and localization were analyzed. Western blot analysis revealed a reduction in Sp1 level in JEG-3 cells treated with TSA, whereas KLF6 whole-cell content was not affected (Fig. 5A). Epifluorescence imaging microscopy suggested that nuclear Sp1 localization was maintained, while KLF6 nuclear localization increased in TSA-treated compared to control cells (Fig. 5B and Fig. 5C). Subcellular fractionation assays confirmed these results. They showed a similar nuclear and a lower cytoplasmic/membrane content of Sp1, and a clear nuclear enrichment of KLF6 accompanied by a decrease in its cytoplasmic/membrane content after TSA exposure (Fig. 5D).

In order to analyze whether the acetylation state of Sp1 and/or KLF6 is modulated by HDAC inhibition, JEG-3 cells were independently transfected with each expression plasmid and then, treated or not with TSA. Immunoprecipitation assays with Sp1 or KLF6 antibodies followed by western blot analysis with an anti-acetyl lysine antibody revealed an increase in Sp1 acetylation in response to TSA, while the acetylated-KLF6 level remained unchanged (Fig. 5E).

Altogether, these results suggest that Sp1- and KLF6-mediated activation of *PSG5* promoter could be enhanced by TSA through an increase in both Sp1 acetylation and KLF6 nuclear localization.

### *PSG5* Promoter is Regulated by Transcriptional Co-activators with HAT Activity

**Figure 6:**
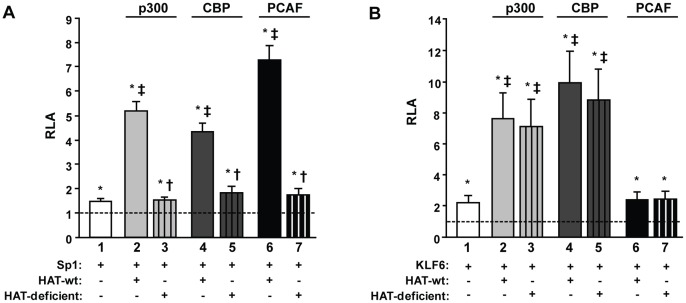
Effect of HAT co-activators on *PSG5* promoter activation mediated by Sp1 and KLF6. JEG-3 cells were transfected with the UB5luc plasmid and the expression vectors coding for Sp1 (A) or KLF6 (B) alone (lane 1) or together with either wild-type (lanes 2, 4, and 6) or HAT-deficient (lanes 3, 5, and 7) versions of p300 (light grey bars), CBP (dark grey bars) or PCAF (black bars). Data are shown as relative luciferase activity (RLA) to that of JEG-3 cultures co-transfected with the corresponding empty vectors defined as 1 (dashed line). Results are presented as mean ± SD of one representative experiment of two independent assays performed in triplicates, with consistent results. Statistical differences (p≤0.05) were identified using two-sided Student’s t-test. * depicts statistically different values compared to that of the culture co-transfected with the corresponding empty vectors. † represents statistically different values compared to that of the culture co-transfected with the wild type version of the corresponding co-activator. ‡ shows statistically different values compared to that of the culture co-transfected with the Sp1 or KLF6 expression vector alone.

CBP, p300, and PCAF proteins are common co-activators for a variety of transcription factors [50], [51]. They have intrinsic HAT activity thus they also mediate acetylation of histone and nonhistone proteins. Furthermore, they can bind and/or acetylate Sp1 and KLF6 [9]–[12], [52], [53]. For these reasons, we next explored the hypothesis that these co-activators modulate Sp1- and KLF6-mediated transcriptional activation of *PSG*. To this end, expression vectors encoding the native or HAT-deficient versions of CBP, PCAF, and p300 co-activators were individually co-transfected together with the UB5luc reporter construct in the presence of overexpressed Sp1 or KLF6. Expression of each co-activator construct was confirmed by western blot assays (data not shown). As shown in Figure 6A, Sp1 stimulation of the UB5luc construct was further increased by p300, CBP, and PCAF (lanes 2, 4, and 6*vs* 1) but not by the HAT-deficient variants (lanes 3, 5, and 7 *vs* 1). On the other hand, p300 and CBP, but not PCAF, were able to further induce the KLF6-dependent UB5luc construct activation (Fig. 6B, lanes 2, 4, and 6 *vs* 1), and HAT-defective p300 and CBP constructs co-activated the *PSG5* promoter to the same extent that their wild type versions (Fig. 6B, lane 3 *vs* 2 and lane 5 *vs* 4). Altogether, these results suggest that CBP, p300, and PCAF modulate *PSG5* expression through a direct acetylation of Sp1 and/or basal machinery components, while CBP and p300 potentiate KLF6-mediated *PSG5* transcriptional activation by a mechanism independent of their HAT function.

## Discussion

Despite the relevance of *PSG* genes for mammalian gestation, the comprehension of the molecular mechanisms that govern their biosynthesis in placental cells is far away from complete. The importance of histone modifications associated with placental-specific gene expression and hence proper placenta differentiation and functioning [54] prompted us to explore the relationship between lysine acetylation and *PSG* gene regulation. In this report we present the first evidence that *PSG* gene expression can be regulated by lysine acetylation involving histone and nonhistone target proteins. We found that PSG transcripts and proteins increase after treatment of placental JEG-3 cells with HDAC inhibitors. TSA not only induces PSG biosynthesis, but more significantly stimulates PSG secretion. HDACis are known to skew the acetylation balance towards an hyperacetylation state affecting chromatin assembly and/or modulating nonhistone protein function [7]. Our results indicate that TSA enhances *PSG* expression through a direct effect on transcription, increasing the amount of acetylH3K9/14 associated with *PSG* promoters. Additionally, it stimulates an augmented Sp1 acetylation and KLF6 nuclear localization that correlate with transcriptional activation of the *PSG5* promoter. Growing evidences point to a relevant role of lysine acetylation in placental-specific gene expression; however the upstream signals that regulate histone or transcription factor acetylation have not yet been identified. For instance, the expression of the trophoblast transcription factor GCM1 depends on deacetylase- and acetylase-mediated regulation [55]. Placental increased expression of *maspin*, a tumor suppressor gene, is associated with a transcriptionally active chromatin characterized by a high level of H3K9 acetylation [56]. Kumar et al. detected an augmented H3K9/14 acetylation and decreased H3K9 methylation linked to the transcriptional induction of the human *CYP19* gene during the differentiation of trophoblast cells [57]. Additionally, transcriptional activation of the *hGH* genes has been linked to unique roles not only for HAT, but also for histone methyltransferase co-activator complexes, and to an expansive histone H3 and H4 acetylation of the locus in syncytium, where the four placental *hGH* genes are expressed at the highest levels [58]. Collectively, these data support our findings suggesting that lysine acetylation may contribute to the remarkable increase of *PSG* gene expression in trophoblast cells. In addition, altogether these observations are in line with the proposal that modulation of HDAC and HAT activities through still unknown upstream biological signals is required for trophoblast differentiation.

Synergistic activation of *PSG5* promoter by Sp1 and TSA suggests that Sp1 acetylation may increase its binding to the CPE element and/or may expose interaction sites to other transcriptional activator proteins to enhance the expression of *PSG* genes. Synergistic activation was also observed by KLF6 and TSA. However, we could not detect any change in the KLF6 acetylation state, but we did observe its clear redistribution into the nucleus. Different scenarios could explain its nuclear enrichment. First, modifications in the activity of a putative cytoplasmic KLF6 partner or a competitor protein could result in KLF6 cytoplasmic release and redistribution to the nucleus. In line with this possibility, recent findings have demonstrated that cytoplasmic c-Src protein interacts with KLF6 and have suggested that KLF6 and estrogen receptor alpha are competitors for c-Src [59]. Second, TSA-induced hyperacetylation could affect other modifying enzyme functionality, such as kinases, which in turn would modify KLF6 allowing its nucleo-cytoplasmic shuttling. Supporting this notion, it has been widely described that HDACis can modulate different kinase signaling pathways. For instance, Cao and coworkers [60] have established that acetylation of MAPK phosphatase-1 blocks the MAPK signaling cascade after TSA exposure. TSA has also been demonstrated to inhibit ERK activation in human renal tubular epithelial cells [61]. Additionally, NaBu has been shown to produce down-regulation of ras/raf/MEK/ERK signaling pathway [62]. Several other reports have documented that HDACis can inhibit PI3K/Akt pathway in diverse cell types [63]–[65]. A third possibility is that the increased nuclear KLF6 localization is due to a raise in its protein expression level. In this sense, it has been reported that KLF6 overexpression in JEG-3 cells results in an enhanced nuclear localization [19]. However, we can rule out this explanation since KLF6 whole cell content was not affected after TSA treatment. Further studies should be performed to identify the precise molecular mechanisms underlying the KLF6 nuclear re-localization induced by HDAC inhibition in trophoblast cells.

The transcriptional co-activators p300, CBP, and PCAF play important roles in multiple biological events including cell growth and differentiation. They enhance transcription by physically linking different regulatory molecules, and their intrinsic HAT activity facilitates transcriptional activation through direct acetylation of histone and nonhistone proteins [66], [67]. Herein, we demonstrated that PCAF, p300, and CBP enhanced Sp1-dependent *PSG5* promoter activation through their HAT functions. On the contrary, we found that p300 and CBP acetyltransferase function was dispensable for sustaining co-activation of *PSG5* promoter by KLF6. Our findings are in line with results of Suzuki *et al*. [68], who demonstrated that p300 binds to and acetylates the Sp1 DNA binding domain, but not that of KLF6. Interestingly, other Sp/KLF family members have been also described to interact differentially with these co-regulators. Particularly, CBP/p300 and PCAF function as Fetal Krüppel-like factor 2 co-activators although only PCAF requires its HAT activity, whereas CBP in a HAT-dependent way, but neither PCAF nor p300, acts as Erythroid Krüppel-like factor co-activator on *gamma-globin* promoter in erythroid cells [69]. Further studies of how co-activators modulate the activity of transcription factors of the same family may improve our understanding of tissue and developmental stage-specific expression not only of *PSG*, but also of other important placental specific genes.

Recent data propose that the final outcome of KLF6 expression depends on the particular cell context, endogenous or external signals, interaction with specific transcriptional partners and/or its subcellular distribution [70]. KLF6 has been shown to be acetylated and to interact with CBP and PCAF in prostate cancer cells *in vivo*, contributing to the up-regulation of *p21^WAF1/cip1^* gene expression [11]. Interestingly, Li *et al.* identified that KLF6 interacts with HDAC3 to achieve *Dlk1* promoter repression, leading to the induction of key regulators of adipocyte differentiation [71]. Nevertheless, in the context of trophoblast cells little is known about the biochemical requirements for KLF6 transcriptional activity and the complexes through which KLF6 regulates target gene expression. As mentioned above, we demonstrated that KLF6 is acetylated in JEG-3 cells and its acetylation state remained unmodified after TSA exposure. Moreover, considering that CBP and p300 functioned as KLF6 co-activators in a manner independent of their HAT activity, it is possible to postulate that KLF6 acetylation is achieved by acetyltransferases other than CBP, p300, and PCAF in this cell context.

Despite *PSG* gene members share a high degree of sequence identity, including their close promoter sequences, disparity expression level has been observed between different human *PSG*s [34], [72], [73]. It has been proposed that subtle nucleotide variation in *PSG* gene promoters allows for differential expression [74]. However, a direct correlation between similar *PSG* transcript level in syncytiotrophoblast and sequence conservation of putative regulatory elements could not be established [34]. In the present study we found that *PSG3* and *PSG5* promoter constructs were differentially activated following HDAC inhibition, suggesting that acetylation might contribute to differential *PSG* gene expression.

In summary, we provided for the first time evidence that lysine acetylation regulates *PSG* expression in concert with Sp1 and KLF6 transcription factors. HDAC inhibition induced *PSG* transcription and protein synthesis correlating with a higher level of acetylated histone H3 associated with the proximal regulatory region of *PSG* genes. We identified different ways to achieve a synergistic *PSG5* promoter activation through TSA and Sp1 or TSA and KLF6. While Sp1 is a direct molecular target of acetylation, KLF6 exhibits a nuclear enrichment after HDACi exposure. Finally, we defined that co-activators with HAT function cooperate in *PSG* transcriptional activation through both Sp1 and KLF6 transcription factors employing mechanisms with differential dependence of their HAT activity. Due to the high nucleotide identity between the different *PSG* genes, it is possible to hypothesize that other family members share similar regulatory mechanisms to the ones identified here for *PSG5.*

Altogether, our findings lead to a better understanding of the molecular basis that control *PSG* expression and open avenues to pursue future research on the molecular mechanisms of *PSG* gene activation during placental differentiation.
